# Electrical Capacitance Volume Tomography: Design and Applications

**DOI:** 10.3390/s100301890

**Published:** 2010-03-09

**Authors:** Fei Wang, Qussai Marashdeh, Liang-Shih Fan, Warsito Warsito

**Affiliations:** William G. Lowrie Department of Chemical and Biomolecular Engineering, the Ohio State University, 140 West 19th Avenue, Columbus, OH 43210, USA; E-Mails: wangf@chbmeng.ohio-state.edu (F.W.); marashdeh.1@osu.edu (Q.M.); wsito@yahoo.com (W.W.)

**Keywords:** electrical capacitance volume tomography, ECT, ECVT, capacitance sensor, multi-phase flow, non-intrusive testing, process imaging

## Abstract

This article reports recent advances and progress in the field of electrical capacitance volume tomography (ECVT). ECVT, developed from the two-dimensional electrical capacitance tomography (ECT), is a promising non-intrusive imaging technology that can provide real-time three-dimensional images of the sensing domain. Images are reconstructed from capacitance measurements acquired by electrodes placed on the outside boundary of the testing vessel. In this article, a review of progress on capacitance sensor design and applications to multi-phase flows is presented. The sensor shape, electrode configuration, and the number of electrodes that comprise three key elements of three-dimensional capacitance sensors are illustrated. The article also highlights applications of ECVT sensors on vessels of various sizes from 1 to 60 inches with complex geometries. Case studies are used to show the capability and validity of ECVT. The studies provide qualitative and quantitative real-time three-dimensional information of the measuring domain under study. Advantages of ECVT render it a favorable tool to be utilized for industrial applications and fundamental multi-phase flow research.

## 1. Introduction

Industries, such as pharmaceuticals, food, and petrochemicals, employ multi-phase flows in their processes. Phases in such processes include gas-solid, gas-liquid, and gas-liquid-solid [1–6]. An insight into phase interactions is essential to the understanding of the operation of multi-phase flows. Such insight is provided by different measurement techniques with quantitative local and global dynamic information of the flow that is useful for system design and control. The area of multi-phase flow measurements has been an intensive research topic for several decades. Accurate multi-phase flow measurement technologies present practical challenges and continued progress is being made toward improving these technologies.

Simple and low cost intrusive probes have been used in many operation systems to obtain flow information [7–16]. They require some penetration in the processes’ flow field for signal acquisition, but such an invasive procedure may interrupt the local physical flow behavior of the flow and affect the accuracy of the measurements. The need for a more accurate measurement technology led to the development of process tomography, a measurement technology that provides cross-sectional images of phase concentrations [17,18]. Process tomography becomes even more appealing when non-intrusive sensors are used to obtain the cross-sectional images. Applications of such process imaging technologies have been gaining momentum over the past several years. Examples include Magnetic Resonance Imaging (MRI), Positron Emission Tomography (PET), X-ray tomography, γ-ray Tomography (GRT), Electrical Magnetic Tomography (EMT), and Electrical Capacitance Tomography (ECT) [19–40]. Those tomography techniques mainly differ in the sensors used and signal obtained to form an image. A particular tomography technology is favored over another for a certain application based on cost, acquisition speed, image reconstruction speed, safety, simplicity, resolution, applicability to vessels with complex geometries and different sizes, 3D capability, and the nature of the processes under investigation. ECT is among the most favored tomography technologies in terms of cost, speed, safety, applicability to vessels of various sizes and geometries, and simplicity. With the recent introduction of volume technology (ECVT), capacitance sensors can now be used for real-time 3D volume imaging [41,42]. Capacitance volume tomography is based on reconstructing a 3D image from a capacitance signal obtained from a 3D capacitance sensor [42]. ECVT utilizes the nonlinearity in electric field distribution to track changes in phase distributions.

ECT was first introduced in the 1980s by a group of researchers from the US Department of Energy Morgantown Energy Technology Center (METC), to measure a fluidized bed system [43,44]. Subsequently, this technology was applied to measure oil concentrations and pneumatic conveyors by a research group led by Maurice Beck at the University of Manchester Institute of Science and Technology (UMIST) during the 1990s [18,32,45–47]. ECT was also applied to image pharmaceutical fluidized bed dryers and three-phase chemical engineering reactors in this same decade [31,48–50]. In solids pneumatic conveying systems and oil pipelines, the cross-sectional components’ holdup distributions are usually obtained from ECT to monitor the flow conditions inside transport vessels [51,52]. In fluidized bed reactors, the gas/solid, gas/liquid/solid holdup, and bubble size distributions are obtained by ECT for basic multi-phase flow science, reactor design, and operation control [16,34,35,38–40,53–58]. Correlations between ECT concentration images from different locations are used to provide the flux of components in the pipeline.

The main limitation of ECT technology is in the inadequate spatial resolution it can provide. The measurement resolution is dependent on ECT sensor designs and image reconstruction. The resolution of ECT images can be improved by increasing the number of sensors. However, the highly nonlinear reconstruction problem is still considered the main obstacle to increasing resolution. This problem is even more evident in the ECVT volume technology as the nonlinearity of the problem is increased substantially. ECVT emerged about five years ago from the labs of The Ohio State University. Researchers from the Technical University of Lodz and the University of Manchester recently developed a 3D electrical capacitance tomography for 3D visualization [59,60]. The resolution is expected to improve as research in ECVT gains increased attention [36,37,41,42,61,62]. Despite this resolution limitation, ECVT leads other imaging technologies in terms of real-time 3D feature, applicability to complex geometries, reduced cost, and low profile sensors. In this review, ECVT will be presented in the context of three-dimensional sensors with different sensor configurations, sizes, and geometries. Several case studies are illustrated to demonstrate the potential usefulness of applying ECVT for imaging multi-phase flow systems. All the capacitance measurements reported in this study were obtained using the PTL charge/discharge 12 channel acquisition hardware with the exception of the images given in Figures 10 and 11-b where the EdWar technology AC-based hardware was used.

## 2. Principle of ECVT

**Figure 1.**
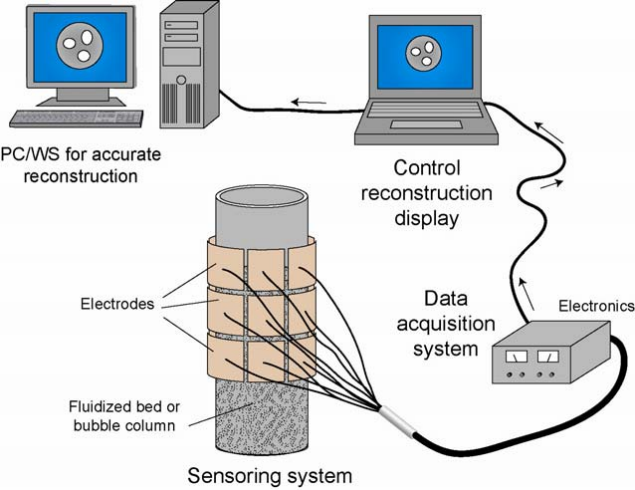
Sketch of an ECVT system.

ECVT technology is based on utilizing nonlinear distributions of electric field lines to reconstruct a volume image of different materials in the imaging domain [42]. The low profile and flexibility of capacitance sensors, increased number of imaging frames per second, and relatively low cost of the ECVT system are characteristics that have moved the technology to the top of the list of industrial imaging tools. This step forward is a continuation to developments in capacitance tomography, a technology that was introduced by the US Department of Energy in the 1980s. Figure 1 is a sketch of an ECVT system.

It consists of an ECVT sensor, data acquisition system, and computer for data recording and image reconstruction. ECVT measures capacitances between all electrode pairs of the ECVT sensor with N channels, which gives a total N (N-1)/2 independent capacitance measurements. The measurement here is a set of normalized independent capacitances from the sensor.

### 2.1. Operating Principle

Similar to other tomography techniques, capacitance tomography in both its 2D and 3D forms is based on reconstructing a physical property from a set of boundary measurements. For capacitance tomography, the physical property is dielectric constant distribution in the imaging domain and the boundary measurement is electric capacitance. Capacitance tomography belongs to the soft field category of tomography techniques where electric filed lines are non-linearly related to dielectric constant distributions. This non-linear relation introduces a higher level of complexity that requires powerful reconstruction algorithms which are discussed in the next section. Electrical capacitance is related to dielectric (permittivity) constant distribution. The electric potential and permittivity distributions are described by the Poisson Equation:

(1) $$\varepsilon (x,y,z)\nabla^{2}\phi (x,y,z)+\nabla \varepsilon (x,y,z)\nabla \phi (x,y,z)=0$$

where *ε(x,y)* and *ϕ(x,y)* represent the dielectric constant (permittivity) distribution and the potential distribution, respectively.

In capacitance volume tomography, sensors can be designed to fit any physical geometry. However, this flexibility further complicates electric field distribution resulting in an uneven distribution of resolution in the final reconstructed image. This problem can only be addressed by employing advanced image reconstruction techniques that consider the sensor design for maximizing image resolution. Different approaches for image reconstruction are discussed in the following section. However, the field of image reconstruction is still open for further improvements.

### 2.2. Reconstruction

The non-linear nature of electric field distributions from capacitance sensors has been the main challenge to the technology resolution for both 2D and 3D volume imaging. Two main reconstruction approaches have been employed to address this problem: linear projection and optimization. In the former, the capacitance measurements are iteratively projected through a linearised sensor matrix to yield a concentration image representative of different phase distributions. In the latter, a set of pre-defined measures are optimized iteratively to yield the most probable image for a set of measured capacitances. Iterative linear reconstruction is simple to implement and requires less computational power. However, it is bounded by its low resolution, introduced image artifacts, and lack of reliable quantitative image representation [34]. Optimization reconstruction, on the other hand, is more complex to implement and requires more computational power. Optimization reconstruction provides better image resolution and less image artifacts, and it is more suitable for applications where quantitative information of the flow under investigation is required. These features become more significant when optimization techniques are used to reconstruct volume images from 3D capacitance sensors. To date, images acquired using the 3D neural network optimization reconstruction algorithm (3D-NN-MOIRT) are considered superior relative to other reconstruction methods [29,38]. This optimization algorithm optimizes four objective functions related to the measured capacitances and reconstructed image. The four objective functions are characterized by the mean square error between forward solution of reconstructed image and capacitance data, the smoothness of reconstructed image, the entropy of reconstructed image, and the 3D-2D matching function of reconstructed image. Optimization of the four objective functions is implemented using the Hopfield neural networks. The advantage of using the Hopfield neural networks for optimizing the final reconstructed image is that it preserves a monotonic decreasing error function as iterations proceed. Optimization reconstruction has also the advantage of optimizing functions related to both the measured capacitance and final image. This advantage is a major improvement for eliminating noise and artifacts from the final image. The Hopfield neural network technique does not require training or prior knowledge of the flow. All reconstructed images reported in this paper were obtained using the 3D-NN-MOIRT reconstruction algorithm. Further details of the reconstruction technique are provided in Warsito *et al.* [42]. Table 1 provides a summary of the three major types of image reconstruction techniques for electrical capacitance tomography.

### 2.3. Potential Applications

Applications of ECVT include current applications of capacitance tomography in addition to new applications introduced by ECVT flexible sensor design. Capacitance sensors have also been used to image gas, liquid, and solid multi-phase flow systems. They have also been used to image reactors of different sizes extending from micro-level columns up to columns of 60 inch in diameter [63]. Capacitance sensors have also been demonstrated to image flames and combustion [64]. In the following part of this review, examples of regular and irregularly shaped sensor are used to demonstrate the feasibility of the technology for imaging regions previously considered inaccessible.

## 3. ECVT Sensors

**Figure 2.**
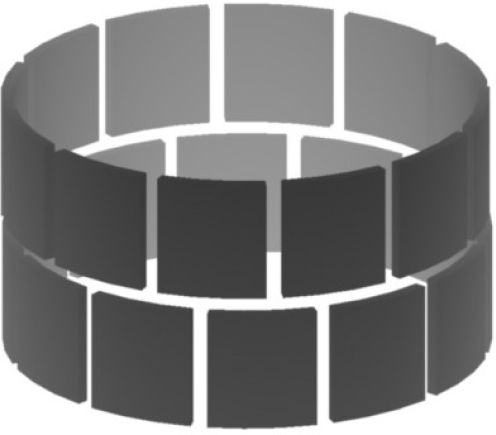
Two-layer ECT sensor configuration.

The sensor shape, configurations, and channel number play an important role both for the two-dimensional ECT and three-dimensional ECVT measurements. Based on the reconstructed image characteristics, capacitance sensors are classified into two-dimensional ECT sensors and three-dimensional ECVT sensors. Figure 2 shows the configuration of a two-dimensional ECT sensor.

It contains two layers and each layer has 12 channels. The two layers are independent and the two-dimensional filed variation in radial directions is realized by measuring the capacitance between a pair of electrodes in the same layer. There are M (M-1)/2 combinations of capacitance measurements in that layer, where M is the number of electrodes in the layer. The length of the ECT sensor in the axial direction is assumed to be infinite here. However, the length of the electrodes in the actual sensor is limited by the sensor dimensions, which is based on practical limitations, leading to what is known as the fringe effect. This effect is translated as artifacts in the final image [42,65]. The permittivity distribution in each test plane is obtained by reconstructing the image from capacitances in the same plane. The permittivity is related to the concentrations of phases, and, as a result, the concentration distribution of the multi-phase flow in the cross-sectional area of the testing vessel is visualized. Du *et al.* [35] used ECT to image solids distribution in the cross-sectional area of the testing vessel for a gas-solid fluidized bed system. Quasi-3D images are usually used in ECT measurement to illustrate the dynamic multi-phase flow behaviors in the testing vessels. A quasi-3D image is a series of two-dimensional images stacked along the z axis of the vessel in the time sequence in which the images are captured. Du *et al.* [35] used the quasi-3D images constructed from a series of images over a period of time to capture the bubble size and shape in the gas-solid fluidized bed.

Two-dimensional ECT sensors have been studied intensively in the past several decades [31]. In most previous ECT studies, the fringe effect was ignored or accounted for as a deviation from the ideal response in an ECT sensor. With the introduction of ECVT, the nonlinear distribution of the electric field is utilized for 3D imaging, and ECVT sensors are designed based on this idea. Field variations in both radial and axial directions are considered for proper ECVT sensor design. A relatively identical distribution of the electrical field in all three dimensions is required for high quality sensor design and image resolution. The sensor shape, electrode configurations, and the number of the electrodes are three key elements in three-dimensional ECVT sensor design. Designs of ECVT sensors on vessels with complex geometries and variable sizes are desired in many industrial processes. The multi-phase process under investigation is the basis for determining a desired sensor design.

### 3.1. Cylindrical Shape Sensor

**Figure 3.**
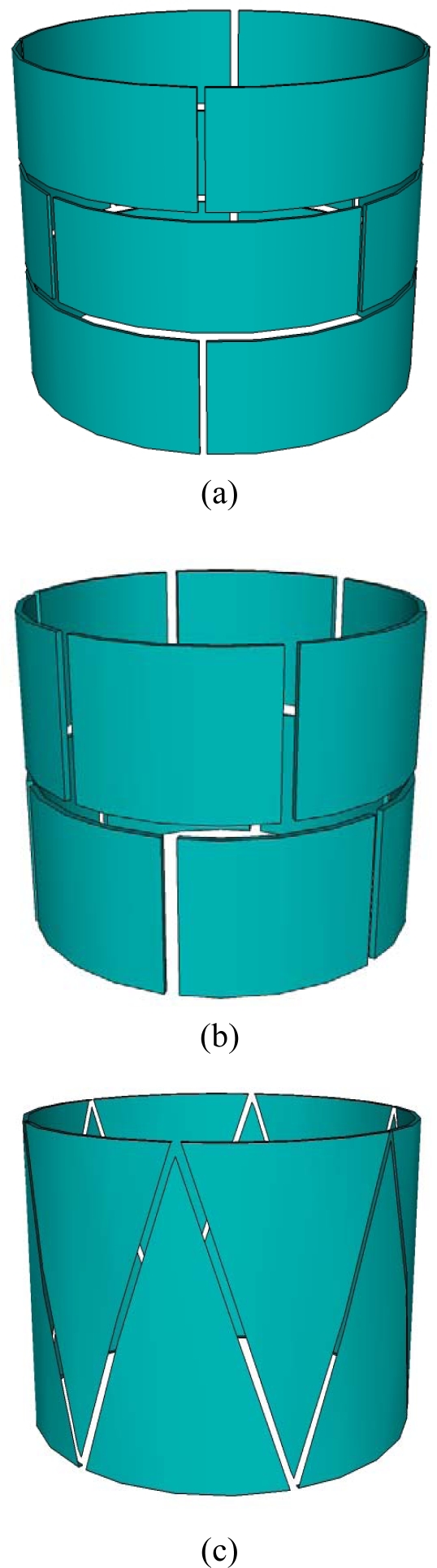
Configuration of ECVT sensor: (a) three-layer sensor; (b) two-layer sensor; (c) single-layer sensor.

Straight vessels, such as fluidized bed reactors, solids pneumatic conveying systems, oil transport pipes, and liquid or gas storage tanks, are widely used in industry [6]. Cylindrical shape ECVT sensors for such vessels are very common due to their regular shape and potential applications in straight vessels. Sensors with multiple layers and rectangular electrodes are typical for cylindrical shape vessels. A variation in the number of layers and plate shape provides control over increasing or decreasing axial resolution with respect to spatial resolution. Figure 3 depicts three different sensor designs for a cylindrical multi-phase flow section. Figure 3(a) is a typical cylindrical shape ECVT sensor. It has three layers and four identical rectangular electrodes in each layer. The electrodes in each layer are shifted 45 degrees with respect to electrodes in adjacent layers. This shift enhances the resolution of the reconstructed image as evidenced by the measurements. The shift is illustrated in Figure 3 (a). The axial and radial resolution increases as a function of the number of layers and the number electrodes in each layer. For a fixed number of electrodes, the increase of layers is countered by a decrease in radial resolution. Varying the electrode shape also plays a role in this resolution trade-off. A limitation on the maximum number of layers that can be used is usually introduced by the data acquisition in operation. The signal to noise ratio for a pair of plates decreases as the separation between plates increases. For applications with limited space in the axial direction, 3D features in ECVT sensors are introduced by changing the shape of the plates rather than increasing the number of layers. Figures 3(b) and 3(c) are some examples of two-layer and single-layer sensors, respectively. The two-layer ECVT sensor illustrated in Figure 3(b) appears to be similar to the two-layer or twin-plane ECT sensor in Figure 2. The difference between them is that the capacitance measurements are also recorded for plates within the same layer and across the layers for the ECVT sensor, whereas the two layers are independent during measurements for the ECT sensor. A useful method to qualitatively asses the quality of the sensor design is to view sensitive regions between each pair of plates. This is accomplished by first forming the sensitivity matrix of the sensor, or solving the forward problem.

**Figure 4.**
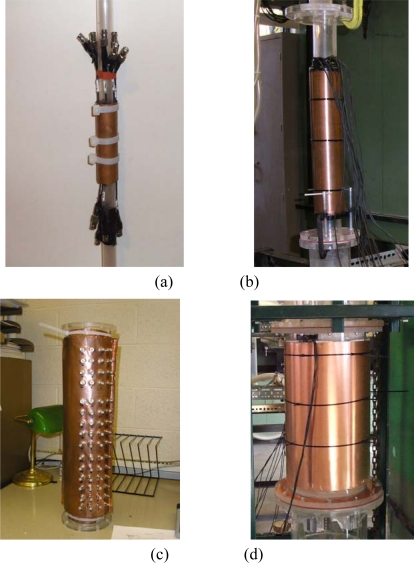
Photos of ECVT sensors with different diameters: (a) 1-in ID; (b) 2-in ID; (c) 4in ID; (d) 12-in ID.

**Figure 5.**
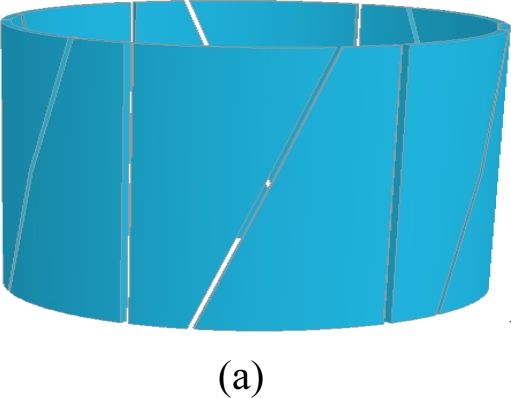

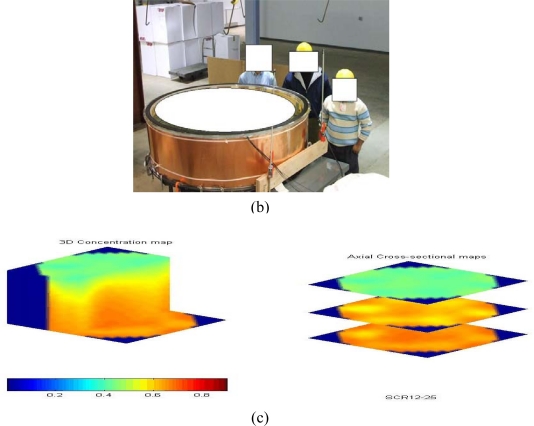
60-in ECVT sensor: (a) sensor configuration; (b) photo of the 60-in sensor; (c) liquid concentration in the unit.

A major advantage of ECVT sensors is that they can be applied to vessels of various sizes. Figure 4 shows an example of sensors with different diameters. A sensor for an upscale unit constructed to image a flow in a 60-in ID vessel is depicted in Figure 5. A triple-layer sensor design may cause radial accuracy problems due to the small length/diameter ratio for the sensing domain of such a 60-in ID vessel. A single-layer ECVT sensor, illustrated in Figure 5(a), which has more electrodes in the layer, is suitable for the measurements. Each capacitance electrode has an inherent 3D characteristic in its design. Figure 5(b) is a photo of the industrial reactor and the 60-in ECVT sensor applied on such system, respectively. The sensor successfully imaged gas-liquid flow in the unit when the unit was running. Figure 5(c) shows the liquid concentration in the unit obtained by the 60-in ECVT sensor.

**Figure 6.**
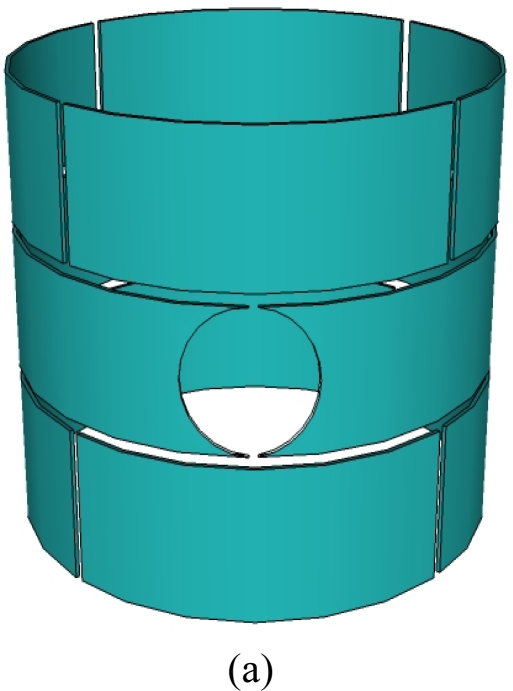

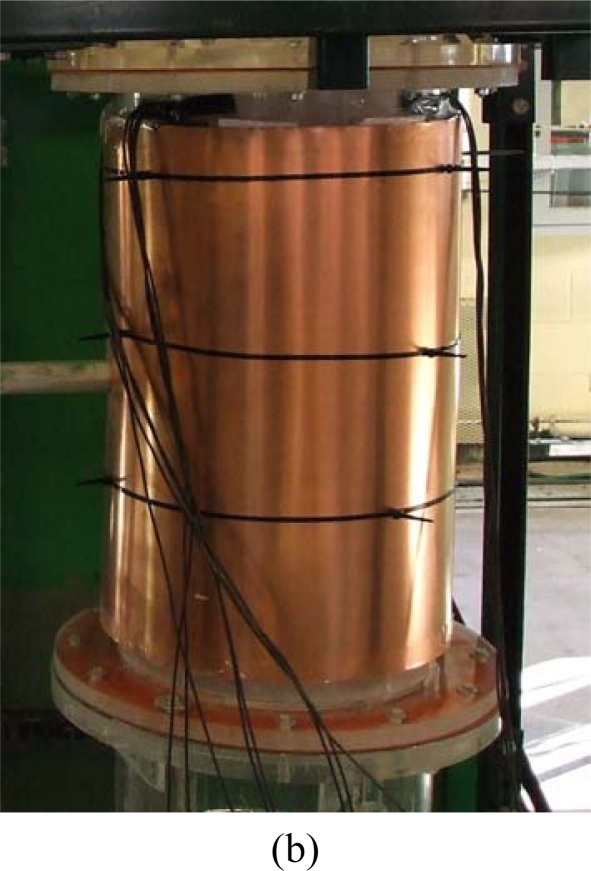
ECVT sensor for the visualization of horizontal gas jet penetration in a gas-solid fluidized bed: (a) configuration of the sensor with a jet tube; (b) photo of the ECVT sensor.

The flexibility of ECVT sensor design is also demonstrated in the modified sensor in Figure 6. In the figure, an ECVT sensor has been successfully applied to visualize instantaneous flow behaviors of a horizontal gas jet into a gas-solid fluidized bed [62,66]. The horizontal gas nozzle is on the wall of the fluidized bed reactor and penetrates the ECVT sensor device. Figures 6(a) and (b) are the sensor design and actual setup of the ECVT sensor modified to accommodate the horizontal gas jet, respectively.

The sensor consists of twelve rectangular electrodes with three layers of capacitance electrodes and four electrodes in each layer. The edges of two adjacent plates in the middle layer of the sensor are modified to fit the horizontal gas tube into the fluidized bed.

### 3.2. Sensors with Complex Geometries

**Figure 7.**
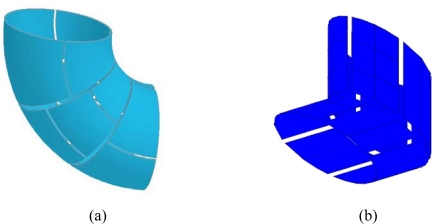

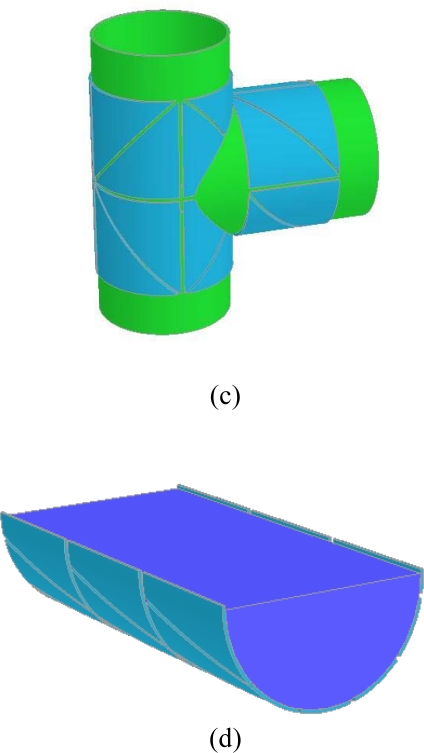
Configurations of ECVT sensors: (a) smooth right-angle bend; (b) sharp right-angle bend; (c) T-shape vessel; (d) half-cylindrical vessel.

Vessels with complex geometries are very common in engineering processes. For example, 90° bends are used to change the flow directions in solids pneumatic conveying systems. T-shape vessels and bends are applied at the inlet and outlet parts in most gas-solid circulating fluidized bed reactors, respectively. The funnel shape is found in the expansion/contraction section of vessels or in reverse-flow cyclones. Flexible ECVT sensors are able to image flows in such geometries by changing the plate shapes to fit the intended geometry. Figures 7(a)–(d) show some ECVT sensor configurations for the smooth right-angle bend, sharp right-angle bend, T-shape vessel, and a half–cylindrical vessel.

**Figure 8.**
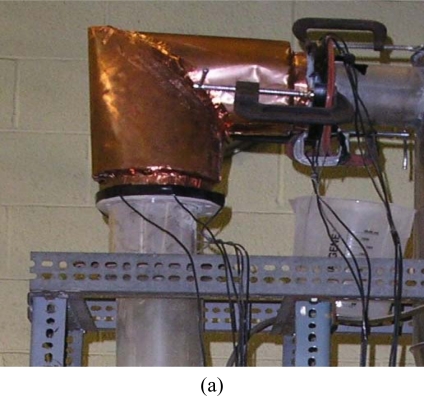

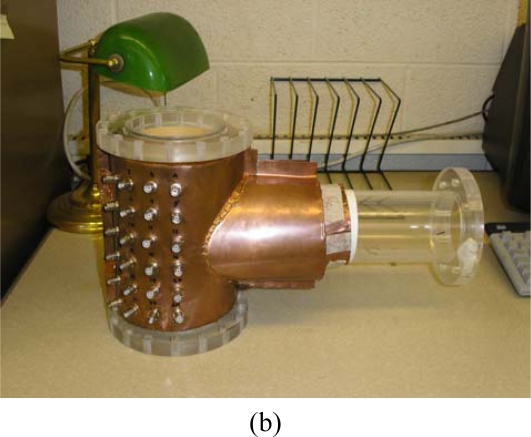
Photos ECVT sensors: (a) right-angle bend sensor; (b) T-junction sensor.

Figure 8 depicts an implementation of a right-angle bend sensor and a T-junction sensor. The image reconstruction process for ECVT sensors with complex geometries follows the same principles discussed in the image reconstruction section.

**Figure 9.**
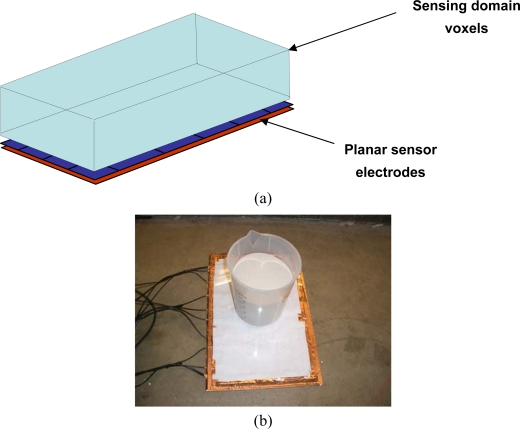

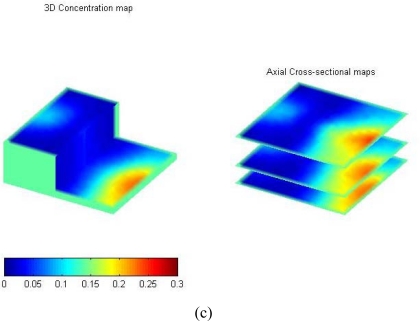
Planar ECVT sensor: (a) configuration of the sensor; (b) photo of the planar sensor with a testing object; (c) tomographic image from the planar sensor.

A planar ECVT sensor has applications related to imaging surface flows. Figure 9(a) shows the configuration of a planar ECVT sensor where all the electrodes are placed on a plane. The measurement domain of the planar sensor is a volume of a cuboid above the plane illustrated in Figure 9(a). Figures 9(b) and 9(c) show an implementation of the sensor with a testing object in the measurement domain and the corresponding reconstructed image.

### 3.3. Sensor Channel Number

**Figure 10.**
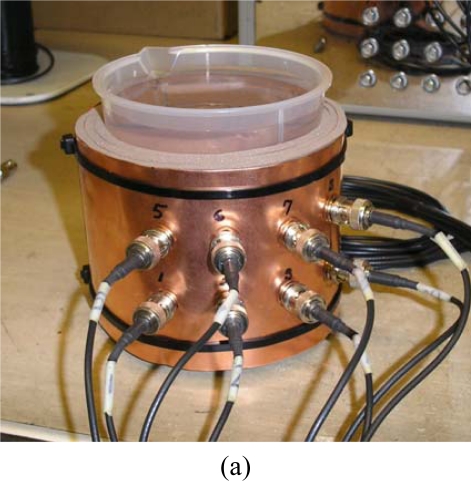

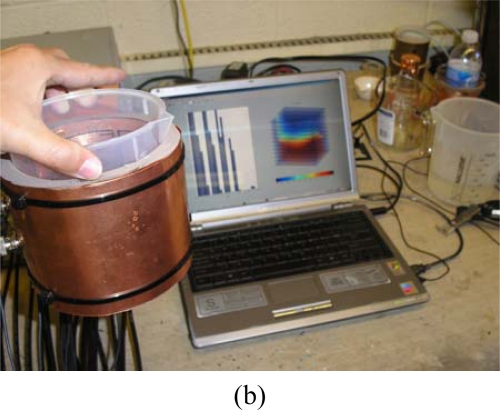
8-channel on-line ECVT system: (a) photo of the 8-channel sensor; (b) on-line water surface measurement using the sensor.

The number of capacitance sensor channels can significantly affect the capacitance measurements. Increasing the channel number is expected to increase the quality of the images. However, the number of channels is restricted by hardware limitations related to decreased signal to noise ratio. An on-line ECVT system has been successfully demonstrated at the Ohio State University. Figure 10(a) shows a photo of an 8-channel on-line ECVT sensor. It has two layers with four electrodes on each layer. The acquisition box is connected to the laptop by a USB cable. Tap-water is used as the fluid media in the container. The on-line images of the water movement in the container and the fluctuation of the water free surface are obtained instantaneously by the ECVT system, illustrated in Figure 10(b).

**Figure 11.**
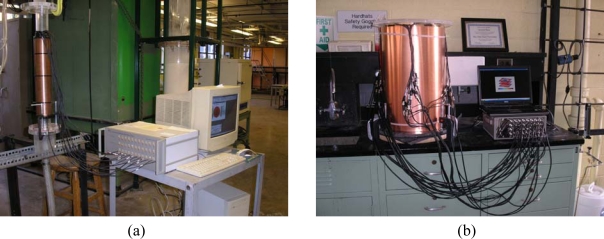
ECVT sensors with multiple channels: (a) 12-channel sensor; (b) 32-channel sensor.

Twelve-channel and 32 channel ECVT sensors, illustrated in Figures 11(a) and(b), were also developed at the Ohio State University.

Different elements of the ECVT system and their effect on the overall operation of the system have yet to be investigated. Increasing the number of electrodes, changing the plate shape, modifying the sensor design, or revising available image reconstruction techniques are all examples of possible developments. Computer simulations are used to design and validate ECVT sensors [65]. They can mimic the capacitance measurements before the fabrication of the real sensor.

The ECVT sensor design is important to the determination of the image resolution and accuracy. In Table 2, several sensor designs are compared based on sensor symmetry, axial resolution, and radial resolution. Three scales, *i.e*., high, moderate, and low are used to describe the quality of the measurement.

## 4. Applications

ECVT has been successfully applied for measurements of multi-phase flow systems. Measurements are provided in real-time for local or global three-dimensional components of multi-phase reactors. The technology has been applied to gas-solid fluidized bed, gas-liquid and gas-liquid-solid bubble columns, gas-solid circulating fluidized beds, and multi-phase flows in vessels with complex geometries. Examples here include imaging of the choking phenomenon, described as a sudden change of dilute solids flow to a slugging flow in the vessel [54]. The experiment took place in a 0.1 m ID gas-solid circulating fluidized bed and a three-layer 12-channel cylindrical 3D ECVT sensor was used. The sensor configuration is described in Figure 3(a). Real-time solids holdup in the riser of the circulating fluidized bed during the choking transition was reported using both a 2D ECT sensor and a 3D ECVT sensor [54]. In another example, Warsito and Fan [41] studied the dynamics of spiral bubble motion in a gas-liquid bubble column with air and Norpar 15 as the gas and liquid phases, respectively. In this experiment, ECVT was used for the measurement of 3D gas and liquid holdups and the dynamic motion of bubble plumes in the bubble columns. ECVT was also used for imaging non-uniform geometries as reported by Wang *et al.* [62,66]. Their work studied the horizontal gas jet penetration in a 12-in gas-solid fluidized bed using a modified cylindrical ECVT sensor. The configuration and real photo of the sensor are illustrated in Figures 6(a) and 6(b). The sensor was used to image instantaneous three-dimensional jet shape as well as developments in the gas-solid fluidized bed. The maximum penetration length and width of the horizontal jet and the coalescence of the horizontal jet with a bubble rising in the fluidized bed were qualitatively and quantitatively investigated using ECVT.

NASA, Tech4Imaging LLC, and National Institute of Standards & Technology are collaborating on a project to use ECVT technology for measurements of a cryogenic fuel gauge in a zero gravity environment [67]. The technology will be assessed for applications for missions in space [68]. ECVT results have been validated by other intrusive and non-intrusive techniques, such as optical fiber probe, pressure transducer, X-ray tomography, magnetic resonance imaging (MRI) and ECT [22,65]. Holland *et al.* [22] quantitatively compared the flow dynamics in a 2 inch ID gas-solid fluidized bed using MRI and ECVT. The average solids holdup, the solids holdup distribution, dynamic bubble shape, motion and bubble frequency on the same system were both obtained from MRI and ECVT. The results from the two techniques were found to be in good agreement. The fast imaging speed of ECVT also enabled the use of 3D images to estimate average voxel velocity [69–72].

**Figure 12.**
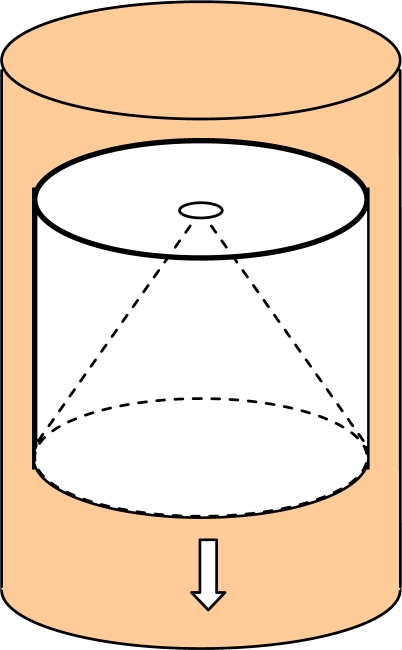
Sketch of a plexiglass cylinder with a conical cavity moving in a cylindrical ECVT sensor.

**Figure 13.**
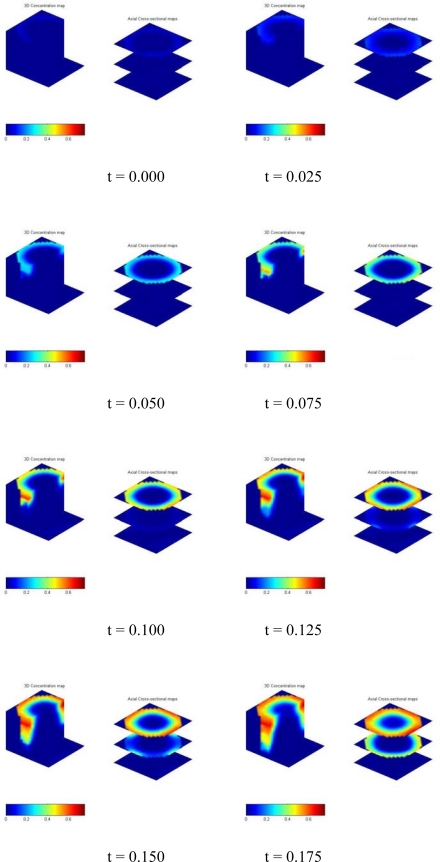

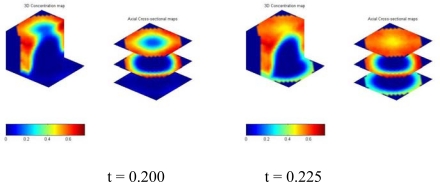
Three-dimensional dynamic view of the cylindrical object moving in the vessel from ECVT.

As a demonstration of ECVT accuracy, a conical cavity in a plexiglass cylinder was imaged moving in a 0.1 m ID vessel, as depicted in Figure 12. The OD and height of the cylindrical object was 10 cm and 13.5 cm, respectively. The ECVT sensor was mounted on the periphery of the vessel. The sensing domain was a cylindrical volume with a diameter of 10 cm and a height of 12.5 cm. 3D NN-MOIRT was used for the image reconstruction with a resolution of 20 × 20 × 20 for the reconstructed images. The acquisition speed was 80 Hz. The phase concentration (Plexiglass) was calculated from the permittivity distribution in the sensing domain based on the capacitance measurements from the wall of the vessel. Figure 13 shows the three-dimensional dynamic view per two frames of the cylindrical object moving downward in the vessel. In each sub image, the left images are snapshots of the x-z plane and half top and bottom x-y planes whereas the right images are snapshots of top, middle and bottom x-y planes of the sensing domain (x, y: two horizontal directions; z: vertical direction). The red and blue colors represent high and low phase concentrations (Plexiglass and air), respectively. The inner conical shape surface, the out cylindrical shape surface and the dynamic positions of the object are clearly illustrated by the three-dimensional images. The results show the capability of ECVT technology to provide real-time, three-dimensional images of complex solid objects in the sensing domain, which can be extended to object detection and monitoring in industrial processes.

**Figure 14.**
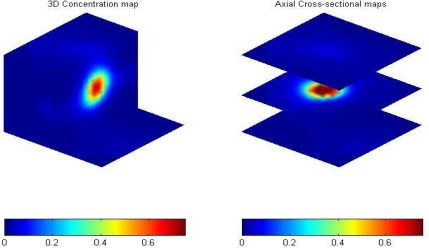
Snapshot of a ball falling in Paratherm fluid by ECVT.

The second example is a ball falling in Paratherm fluid. A ping pong ball with a diameter of 3 cm filled with glass beads with a density of 2500 kg/m^3^ and an average diameter of 250 μm was initially held by a thread statically in Paratherm fluid with a density of 867.9 kg/m^3^ and a viscosity of 0.031 Pa*s contained in a 0.1 m ID column mounted with a cylindrical ECVT sensor. The ball was left for free-fall with a zero initial velocity. The process of the falling ball in Paratherm fluid is obtained by ECVT and a snapshot of the ball in the liquid is illustrated in Figure 14.

**Figure 15.**
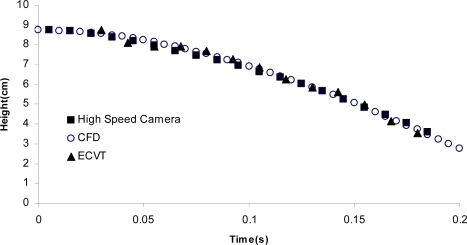
Comparisons of measurements using high speed camera, ECVT, and CFD for the relative position and time of a free falling ball in the Paratherm fluid.

Red and blue colors represent solids and fluid, respectively. The vertical position of the center of the ball is plotted against time using three different methods, ECVT (▴), computational fluid dynamics-CFD (○), and a high speed camera (▪) in Figure 15. The high speed camera experiment was conducted with identical conditions to the ECVT experiment in a transparent column. A Photron FASTCAM PCI high-speed CCD camera with 500 FPS was used to capture the position of the ball center in the column. A CFD model using the fraction volume method was used to track the motion of the ball in the fluid. The method is based on the two-way coupling technique that uses a cubic mesh in the Cartesian coordinates [73]. The particle size is about 15 times that of the mesh size. The velocity in each computational cell is represented by the volume-weighted average of the fluid velocity and particle velocity. The fractional step algorithm is employed in the solution procedure, in which an intermediate fluid velocity is calculated first according to the Navier-Stokes equation, and then the flow field is corrected by considering the fluid-particle interaction in each cell. The numerical method has been implemented based on the CFDLIB code, which was originally developed by Los Alamos National Laboratory, but has been extensively modified by Fan [74] to model various multi-phase problems. The position of the ball in Figure 15 verifies the accuracy of ECVT for tracking differences in phase concentration, as the results from ECVT, the high speed camera and CFD were found to be in very good agreement.

**Figure 16.**
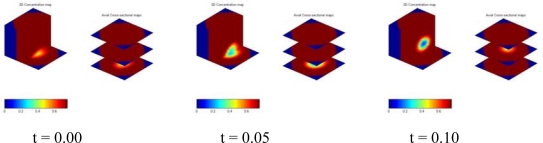

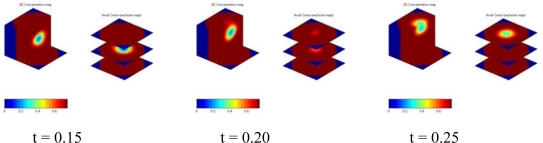
Snapshots of a single bubble rising in a bubble column with Paratherm fluid by ECVT.

**Figure 17.**
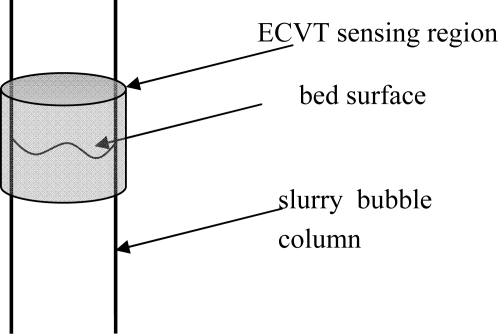
Sketch of an ECVT sensor monitoring the bed surface in a slurry bubble column.

**Figure 18.**
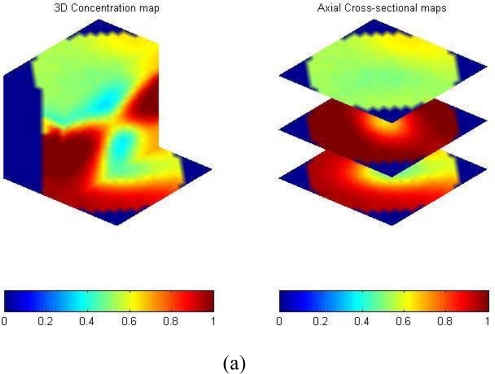

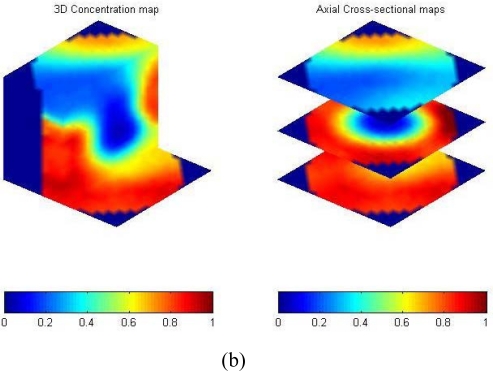
Snapshots of bubble bursting at the surface of a slurry bubble column by ECVT: (a) 3D image before bubble bursting; (b) 3D image after bubble bursting.

Figure 16 illustrates a single bubble rising in a bubble column with Paratherm fluid. A certain amount of air was first held in a cap at the bottom of the sensing domain. The cap was turned over and a single bubble formed above the cap and rose upward. The bubble kept its shape during the motion. Another example is the bubble bursting process at the bed surface in a gas-liquid-solid slurry bubble column [71]. Air, Paratherm fluid, and alumina oxide-based particles with a mean diameter of 150 μm and a density of 1500 kg/m^3^ were used as the fluidizing gas, liquid phase, and solid phase, respectively. The solids loading in the slurry was 9.1 vol.%. A superficial gas velocity of 0.2 m/s was used in the slurry bubble column. The ECVT sensor was mounted near the bed surface area outside the column to image the bed surface in the ECVT sensing domain, as illustrated in Figure 17. Figure 18 depicts 3D images before and after a bubble bursting through the surface of the slurry bubble column. After bursting, more liquid and solids were pushed upwards to the freeboard and to the wall, and a cavity with high gas fraction was formed at the surface of the bed. Small film and jet droplets formed during the bubble bursting were not detected by ECVT due to the volume image resolution of 5 mm × 5 mm × 6 mm.

**Figure 19.**
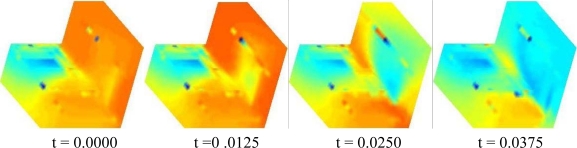

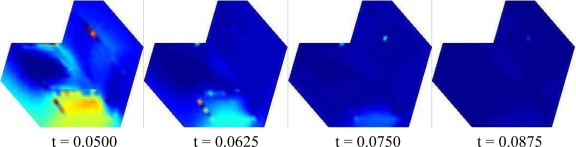
Snapshots of solids discharge in a 90° bend by ECVT.

Tomography measurements of complex geometries are rarely reported due to the obscure sensor design and the complexity of implementing image reconstruction. However vessels with complex geometries are very common in engineering processes. Images of multi-phase flows in such vessels are desired in research and for industrial reactor designs. Here, three-dimensional ECVT imaging of solids flow in a 90° bend is the first to be reported, demonstrating the capability of ECVT sensors for imaging flows in vessels with complex geometries. The sensor configuration and a photo of the sensor are provided in Figure 7(b) and Figure 8(a), respectively. The outlet of the bend is 45 degrees facing downward to the ground and a plate was installed to block particles from falling. FCC particles (Group A) with a density of 1400 kg/m^3^ and a mean diameter of 60 μm were poured in the bended vessel. The sensor used here is similar to the one depicted in Figure 7(b). After the plate was removed, FCC particles were discharged from the bended vessel by gravitational force. Figure 19 shows the dynamic images of the solids concentration in the bend during the discharge process. Red and blue colors represent high and low FCC solids concentrations, respectively. Quantitative images from the ECVT bended sensor can be used to study the hydrodynamics of gas-solid flow in the elbow of solids pneumatic transport vessels and in the exit region of gas-solid circulating fluidized beds.

## 5. Conclusions

In this article, recent advances and progress of electrical capacitance volume tomography are described. The 3D ECVT sensor design variability coupled with a robust image reconstruction algorithm render it possible to obtain, in good quality, the real-time, three-dimensional images of multi-phase flows in the sensing domain from capacitance measurements. The ECVT sensor design is based on the properties of the nonlinear distribution of the electric field for 3D imaging and field variations in both radial and axial directions. The sensor shape, electrode configurations, and the number of the electrodes are the three key elements in 3D ECVT design. ECVT sensors can be applied to vessels of various sizes. An upscale ECVT sensor was constructed to image a 3D gas-liquid flow in a 60-in ID industrial reactor. ECVT sensors are flexible and can be modified for more specific applications. The modification of an ECVT sensor for the measurement of a horizontal gas jetting phenomenon in a gas-solid fluidized bed was presented. In addition to the 3D feature, ECVT has the advantages over other imaging technologies in the applicability to complex geometries such as bends, T-junctions, and funnels. Several case studies that illustrate the capability and validity of ECVT sensors and provide qualitative and quantitative real-time three-dimensional images of multi-phase flows in vessels are presented in this article. The advantages of this technique that allow ECVT to be utilized for general applications for fundamental research and industrial-scale measurements of multi-phase flows in complex multi-scale vessels are also given.

## Figures and Tables

**Table 1.** Key techniques used in solving the inverse problem of capacitance tomography.

**Reconstruction**	**Methodology**	**Characteristics**	**Example**
Single Step Linear Back Projection	The sensor system is linearized (usually by constructing a sensitivity matrix). The image is obtained by back projecting the capacitance vector using the sensitivity matrix.	Fast, low image resolution, and introducing image artifacts	LBP [33]
Iterative Linear Back Projection	The mean square error between the capacitance data and forward solution of the final image is minimized by iterative linear projections using the sensitivity matrix.	Slower than Single Step Linear. Providing better images than Single Step [33]	Landweber ILBP [33]
Optimization	A set of objective functions are minimized iteratively to provide the most likely image. Different optimization algorithms and objective functions can be used.	Slower than Iterative Linear Back Projection. Providing better images than Iterative Linear Back Projection [42]	3D-NNMOIRT [42]

**Table 2.** Comparison of different sensor geometries in terms of symmetry, axial resolution and radial resolution.

**Sensor Type**	**Sensor Symmetry**	**Axial Resolution**	**Radial Resolution**
**Cylindrical sensor with 1 layer**	High	Low, sensitivity decreases toward center.	High, sensitivity decreases toward center.
**Cylindrical sensor with 2 shifted layers**	Moderate	Moderate, sensitivity decreases toward center.	Moderate, sensitivity decreases toward center
**Cylindrical sensor with 3 shifted layers**	Moderate	High, sensitivity decreases toward center.	Moderate-High, sensitivity decreases toward center.
**Planar sensor with shifted planes**	Moderate	Low, sensitivity decreases away from sensor.	High, Sensitivity decreases away from sensor.
**Bent sensor**	Low	Depends on sensor plate arrangement	Depends on sensor plates arrangement

## Nomenclature

**Notations**
*M*: number of electrodes in the layer of an ECT sensor
*N*: number of channels in an ECVT sensor
*x,y*: two horizontal directions in the sensing domain of a cylindrical sensor
*z*: vertical direction in the sensing domain of a cylindrical sensor

**Greek letters**
*ε*: dielectric constant (permittivity) distribution in the sensing domain
*ϕ*: potential distribution in the sensing domain
